# Characterizing Spinal Decompression for Foot Drop Caused by Lumbar Degenerative Disease: A Systematic Review and Meta-Analysis of Cohorts

**DOI:** 10.3390/jcm14134470

**Published:** 2025-06-24

**Authors:** Christian A. Than, May Y. Hajeir, Lamees M. Al Darwashi, Kelly Silnes, Aslam Mohamed Haroon, Angelique K. Valiotis, Diana Shibib, Yasmine J. Khair, Hugh Milchem, Persidiu Iancu, Zaher Dannawi

**Affiliations:** 1 Cambridge University Hospitals NHS Foundation Trust, Cambridge CB2 0QQ, UK; 2 School of Biomedical Sciences, The University of Queensland, St Lucia, QLD 4072, Australia; 3 University of Nicosia Medical School, University of Nicosia, 2417 Nicosia, Cyprus; 4 Faculty of Medicine, University of Buckingham Medical School, Buckingham MK18 1EG, UK; 5 St George’s University of London, London SW17 0RE, UK; 6 Mid and South Essex NHS Foundation Trust, Essex SS0 0RY, UK

**Keywords:** foot drop, herniation, lumbar degenerative disease, decompression, discectomy, meta-analysis, spine, manual muscle testing

## Abstract

**Background/Objectives**: There exists a need to capture the current landscape of the literature for lumbar decompression on muscle strength, as measured by manual muscle testing (MMT), in cohorts with foot drop secondary to lumbar degenerative disease (LDD). **Methods**: A literature search of PubMed, EMBASE, CINAHL, Cochrane Central Register of Controlled Trials, Cochrane Database of Systematic Reviews, Scopus, and Web of Science was conducted from each database’s inception to 21 March 2025. Eligible studies reported patients with LDD-related foot drop treated surgically. This review was registered in PROSPERO (ID: CRD42024550980). **Results**: A total of 20 studies comprising 918 patients met the eligibility criteria, with most cases attributable to lumbar disc herniation (79% of patients, 95% CI: 0.72–0.85, I^2^ = 96%) or spinal stenosis (22% of patients, 95% CI: 0.15–0.30, I^2^ = 96%). Following surgery, 60% of patients (95% CI: 0.44–0.75, I^2^ = 97%) achieved an MMT score of 4–5, indicating recovery, while 82% (95% CI: 0.76–0.88, I^2^ = 89%) demonstrated an improvement of at least one MMT grade. No improvement was seen in 18% of patients (95% CI: 0.12–0.24, I^2^ = 89%). For pain, the preoperative VAS mean was 5.91 (95% CI: 4.21–7.60, I^2^ = 99%), while the postoperative mean was 1.00 (95% CI: −0.05–2.06, I^2^ = 99%). Overall complications were reported at 1% (95% CI: −0.00–0.02, I^2^ = 0%). **Conclusions**: Lumbar decompression achieves clinically meaningful recovery of LDD-induced foot drop. However, this meta-analysis highlights the overlooked portion of patients who will not respond, providing a sequential approach for future investigation of these cohorts through foundational evidence of the present literature base.

## 1. Introduction

Foot drop is a debilitating condition characterized by the weakness of ankle dorsiflexors [1]. It can arise from a variety of etiologies, including central nervous system disorders, peripheral nerve pathologies (peroneal nerve compression is the most common), systemic diseases, infections, or iatrogenic factors [2]. Lumbar degenerative diseases (LDDs), including lumbar disc herniation (LDH) and lumbar spinal stenosis (LSS), are frequent causes of foot drop, typically through compression of the L5 nerve root [2,3]. It is present in up to 7.7% of patients with LDD [4], with the corresponding gait disturbance significantly impairing mobility. The cost of foot drop-related hospital admissions has been estimated at USD 30,000 per patient [5].

The severity of weakness can be evaluated via the manual muscle test (MMT) of ankle dorsiflexors and graded in accordance with the Medical Research Council (MRC) [6]. MMT scores range from 0 (no contraction) to 5 (against gravity and full resistance) [7]. A score of 3 and below typically indicates foot drop, characterized by the inability of patients to lift their foot against gravity [6]. Lumbar decompression, consisting of discectomies or laminectomies, aims to treat this underlying pathology by directly relieving nerve or spinal cord pressures [8,9]. Thus, lumbar decompression has demonstrated foot drop recovery rates through improved postoperative MMT values of 4 and above, ranging from 27% to 84% [1,10]. It is, therefore, the treatment modality of choice to promote neurological recovery.

Although the prevailing opinion is that decompression should be offered to all patients suitable for surgery, the current literature lacks corroborative encapsulation on success and failure rates. Prior meta-analyses have investigated the timing of surgery [1] and prognostic factors for surgical outcomes with selective evidence available [11,12]. However, to our knowledge, there are limited recent meta-analytic data aimed at characterizing the current literature from higher-level cohort studies. As meta-analyses remain the highest form of hierarchical evidence [13], there exists an academic need to address this gap.

Therefore, this meta-analysis aims to provide a comprehensive pooled snapshot of existing cohorts investigated for lumbar decompression to treat LDD-caused foot drop. The findings here serve to provide foundational evidence on the present state of the literature, which may inform future study designs in targeted understanding of cohorts who do and do not recover after surgical intervention.

## 2. Materials and Methods

### 2.1. Data Search Strategy and Resources 

This review was conducted in accordance with the Preferred Reporting Items for Systematic reviews and Meta-Analyses (PRISMA) statement [14]. A comprehensive search of several databases from each database’s inception to 21 March 2025 was conducted with no language restriction. The databases included PubMed, EMBASE (Elsevier), CiNAHL, Cochrane Central Register of Controlled Trials, Cochrane Database of Systematic Reviews, Scopus, and Web of Science. The Cochrane Database of Systematic Reviews was included to hand search the references of the relevant prior literature. The search terms were created by a medical reference librarian in consultation with the primary investigator. Controlled terms were used to identify studies describing foot drop as a result of LDD, including LDH or LSS, managed with lumbar decompression. The actual strategy listing all search terms used and how they are combined is available in Supplemental Item S1. This review was registered prospectively with PROSPERO (ID: CRD42024550980). 

### 2.2. Inclusion and Exclusion Criteria

Studies were deemed eligible if they met all of the following inclusion criteria: (1) Investigated patients with foot drop resulting from LDD; (2) Used lumbar decompression as the primary treatment; (3) Reported on the primary outcome of MMT based on the MRC grading system; (4) Employed a study design of randomized controlled trials (RCTs), observational cohort studies (prospective or retrospective), or case series. In articles where multiple studies originated from the same institution or authors, the most recent or highest quality study was selected to prevent overlapping data.

Studies were excluded if they met any of the following criteria: (1) Non-LDD caused foot drop, such as peroneal nerve entrapment or malignancies; (2) Pathology involving the thoracic vertebrae or regions above the lumbar spine; (3) Studies reporting conservative treatment methods only without surgical intervention; (4) Literature studies that did not report primary outcomes relevant to the analysis; (5) Unpublished data, data published in abstract form only, or non-full-length articles (6) Review articles, letters to the editor, case reports, conference abstracts, or similar non-original research formats; (7) Articles not available in English, where translations were not accessible.

Article screening was conducted by three independent assessors (AKV, DS, and HAM), with data extraction conducted by three independent assessors (KS, LMFD, and MYHH). Any disagreements were adjudicated by CAT and discussed with co-authors as necessary. The quality of each study was assessed independently by two authors (LMFD and MYHH) using the Risk Of Bias In Non-randomized Studies—of Interventions (ROBINS-I). In cases of disparity, two independent assessors deliberated, and disagreements were settled through adjudication by YJK.

### 2.3. Outcomes

This meta-analysis primarily focused on evaluating the postoperative muscle strength of the ankle dorsiflexors as a measure of surgical success and recovery. Muscle strength was assessed using the manual muscle test (MMT), which is based on the Medical Research Council (MRC) grading system. MMT scores range from 0 (no contraction) to 5 (normal strength against gravity and full resistance) [15]. A definition of foot drop was accepted as ≤ 3 MMT as previously documented in the literature or if explicitly classified by included study authors [6]. Recovery rates were measured, with a score of 4 indicating partial recovery and a score of 5 representing full recovery. In our focus on measuring recovery, MMT scoring was grouped as 4–5 in accordance with available data. Any improvement, but not necessarily partial or complete recovery, of postoperative MMT ≥ 1 was also extracted. Pain levels were assessed as a secondary outcome using the Visual Analogue Scale (VAS) questionnaire, with a scoring scale of 0 (no pain) to 10 (worst pain) [9,16,17,18,19]. Additional secondary outcomes included complications, such as infections and spinal leaks, as well as the number of patients undergoing spinal fusion procedures. Furthermore, re-operation rates, duration from foot drop onset to surgery, and preoperative characteristics, including preoperative imaging, herniation grading, comorbidities, and spinal levels involved, were extracted where applicable. The surgical modality was defined as per the included study author descriptions. Where descriptions were not clear, surgical modality was classified as unspecified decompression. In studies involving patients with bilateral foot drop, the MMT score of the weaker leg was typically reported by the authors [3,15,17,20,21]. When MMT scores of both legs were provided, only the weaker leg’s MMT score was extracted to ensure consistency across studies and to avoid overestimating the surgical effect [22].

### 2.4. Statistical Analyses

The means of continuous variables and rates of binary variables were pooled using the generic inverse variance method of Der Simonian, Laird [23]. Proportions underwent logit transformation prior to meta-analysis. The heterogeneity of effect size estimates across the studies was quantified using the Q statistic and the I^2^ index (*p* < 0.10 was considered significant) [24]. A value of I^2^ of 0–25% indicates minimal heterogeneity, 26–50% moderate heterogeneity, and 51–100% substantial heterogeneity. The random-effects model was used [24]. If the mean and standard deviation (SD) were unavailable, the median was converted to the mean using the formulas from the *Cochrane Handbook for Systematic Reviews of Interventions* [25]. If SD was not available or extractable, the reported mean was omitted from the calculation. Authors were contacted three times to obtain any relevant additional information that was omitted in published articles. Postoperative publication bias was assessed visually using funnel plots [26]. Data analysis was performed using Open Meta analyst software v0.24.1 (CEBM, Brown University, Providence, RI, USA). All included studies were categorized as having one study arm for analysis.

## 3. Results

**Figure 1:**
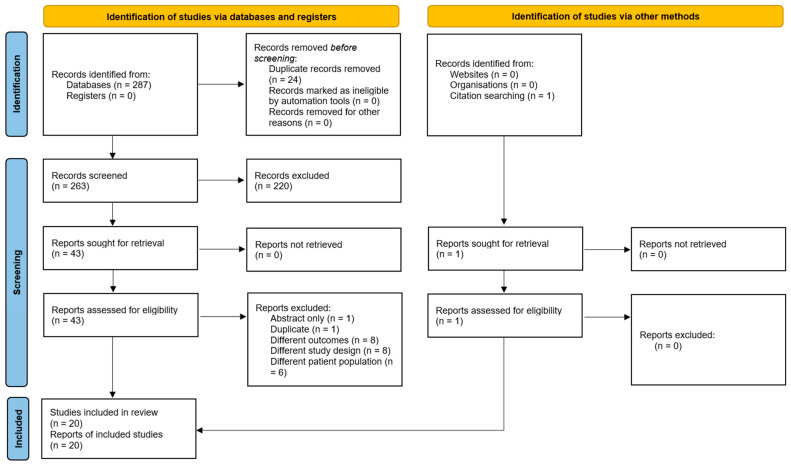
Preferred Reporting Items for Systematic Reviews and Meta-Analyses (PRISMA) flowchart.

The initial search identified 287 potentially relevant studies, 20 of which met the eligibility criteria [2,3,4,8,9,10,15,16,17,18,19,20,21,22,27,28,29,30,31]. A total of 918 patients were included, with a gender division of 59% male (*n* = 538) and 41% female (*n* = 380). A total of 16 studies were retrospective in design, 1 was prospective [18], and 3 were case series [19,21,22]. Of the articles reporting on patient age, the mean age range was from 46.00 years to 66.90 years [2,3,4,8,9,10,15,16,17,18,19,20,21,22,27,28,30,31,32]. The mean follow-up range was 0.03 months to 81.00 months [3,4,9,10,15,16,17,18,19,20,21,22,27,28,29,30,31]. The PRISMA flow diagram outlines the study selection process in Figure 1. Table 1 provides a detailed overview of the clinical characteristics of each included study.

### 3.1. Risk of Bias

**Figure 2:**
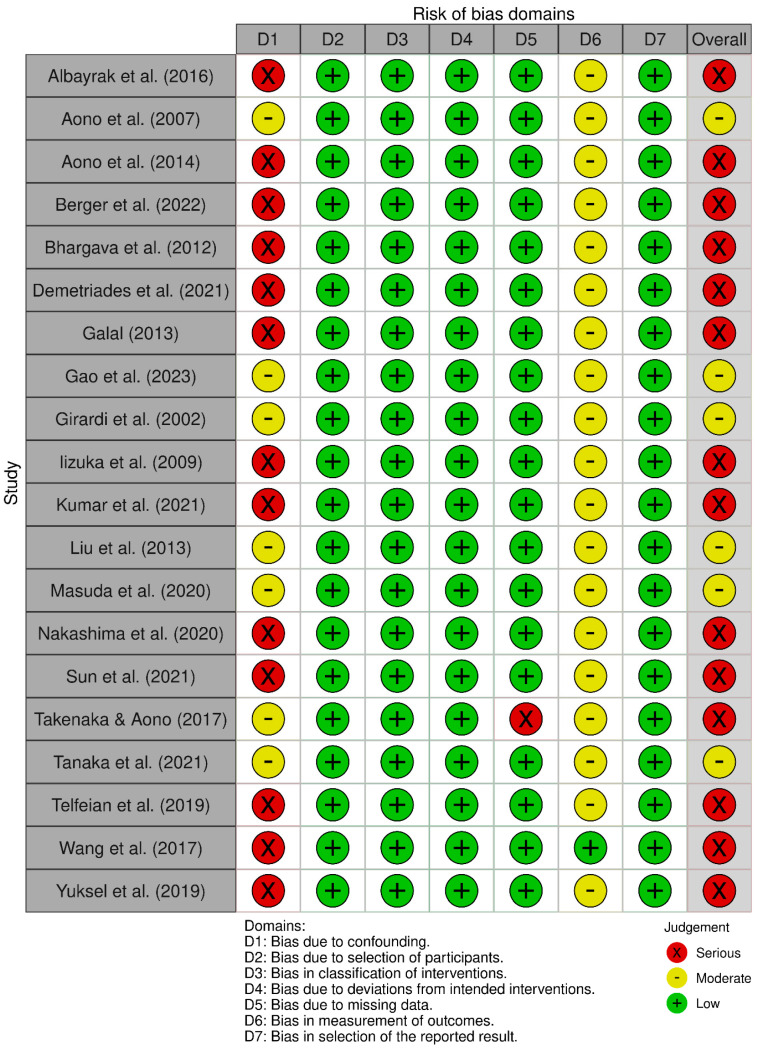
Risk of Bias in Non-randomized Studies—of Interventions (ROBINS-I) assessment [2,3,4,8,9,10,15,16,17,18,19,20,21,22,27,28,29,30,31,32].

The results of the quality assessment of all included studies are displayed in Figure 2. All studies were observational, with ROBINS-I utilized for judgment. A total of 30% (*n* = 6) were considered moderate risk [2,3,16,17,20,27], whilst 70% (*n* = 14) were considered serious risk [4,8,9,10,15,18,19,21,22,28,29,30,31,32]. The exposure and outcomes were appropriately measured, and the follow-up period was sufficient to observe any changes in clinical outcomes. Serious risk was noted in domains of confounding [4,8,9,10,18,19,21,28,29,30,31,32] and of missing data [15].

### 3.2. Preoperative Characteristics

Preoperative characteristics have been reported in Table 2. There were 3 reports of operative levels of L1/L2 [15], 20 reports of operation on L2/L3 [10,15,16,21,27,30], 172 reports of operation on L3/L4 [3,4,9,10,15,16,17,21,22,27,28,30,32], 621 reports of operation on L4/L5 [3,4,8,9,10,15,16,17,21,22,27,28,29,30,31,32], and 202 reports of operation on L5/S1 [3,4,8,9,10,15,16,17,19,21,22,27,28,29,30,31,32]. Out of 918 patients reported for operative levels, 601 were single-level operations [2,3,4,8,9,10,15,16,17,18,19,20,21,22,27,28,29,30,31,32] and 317 were multi-level operations [2,3,4,10,15,16,17,18,20,21,22,27,28,30,32,33]. Out of 706 patients reported for laterality, there were 644 reports of unilateral foot drop [2,3,4,8,9,10,17,19,20,27,29,32] and 62 reports of bilateral foot drop [2,3,8,10,15,17,20,21,22,27,32]. Out of 269 patients reported for preoperative imaging, there were 63 reports of radiography [9,29], 33 reports of CT [9,21], and 268 reports of MRI [3,8,9,19,21,22,28,29,30,32].

Out of 891 patients with reported aetiologies, there were 629 reports of lumbar disc herniation [2,3,4,8,9,10,15,16,17,19,20,21,22,27,28,29,30,31,32], 254 reports of lumbar spinal stenosis [2,3,4,8,10,15,17,20,21,22,27,30,32], 27 reports of spondylolisthesis [3,4,10,15,20,27] and 1 report of spondylolysis [27]. Out of 157 patients reported for herniation classification, there were 5 reports of bulging [22,32], 73 reports of protrusion [8,9,21,32], 35 reports of extrusion [22,29,30,32], and 40 reports of sequestration [29,30,32]. The proportion estimates, confidence intervals, I^2^ values, included study *n* values, and patient sample size *n* values of preoperative characteristics have been summarized from forest plots in Table 2.

Primary surgical modality has been reported in Table 3. Of 918 patients, there were 212 reports of open discectomy [2,3,4,10,15,20,27,30], 198 reports of unspecified decompression [2,17,32], 106 reports of microdiscectomy [8,21,28,29,31], 99 reports of laminectomy/laminotomy [3,20,22,30], 68 reports of fenestration [4,10,15,21,22,27], 37 reports of transforaminal endoscopic discectomy [9,19], 23 reports of tubular discectomy [28], and 2 reports of spondylolysis repair [15,27]. A total of 319 reports of fusion were demonstrated [2,3,4,10,15,16,17,18,20,22,27,30]. The proportion estimates, confidence intervals, I^2^ values, included study *n* values, and patient sample size *n* values of primary surgical modalities have been summarized from forest plots in Table 3.

Comorbidities and impairments can be seen in Table 4. Gluteus medius paralysis and radiculopathy have been referred to as impairment by Berger et al. [28]; hence, these were considered as such in our analysis as well. Out of 433 patients reported for impairments, there were 269 reports of radiculopathy [2,4,8,10,15,21,27,28,29] and 108 reports of gluteus medius paralysis [10,20,21]. Out of 637 patients reported for comorbidities, there were 39 reports of diabetes [2,3,4,22,28,29,30,31,32], 33 reports of ‘other’ comorbidities delineated further into 23 reports of smokers, 6 reports of rheumatoid arthritis, 3 reports of COPD, 1 report of asthma, [2,3,29,31,32], 25 reports of hypertension [2,28,31,32], 9 reports of ischaemic heart disease [2,3,28,32], 4 reports of dyslipidemia [28], 0 reports of osteoporosis, and 0 reports of spinal trauma. Moreover, there were 48 reports of cauda equina [8,10,15,21,27]. The proportion estimates, confidence intervals, I^2^ values, included study *n* values, and patient sample size *n* values of comorbidities and impairments have been summarized from forest plots in Table 4. The forest plots for preoperative characteristics are available as Supplementary Figures S1–S7.

### 3.3. Postoperative Outcomes

MMT scores are outlined in Table 5. Preoperatively, out of 764 patients, there were 326 reports with scores of 0–1 [4,8,10,15,16,17,19,20,21,22,27,29,30,31,32], 421 reports with scores of 2–3 [2,4,8,10,15,16,17,19,20,21,27,29,30], and 17 reports with a score of 4 [2]. MMT of 4 was included from one study only [2] that explicitly classified these patients as having foot drop. Postoperatively, out of 650 patients, there were 109 reports with scores of 0–1 [3,8,9,16,17,20,22,29,30,31] and 210 with scores of 2–3 [2,3,8,9,16,17,18,19,20,29,30,31,32]. No improvement was seen in 149 reports out of 724 patients [3,8,10,16,17,20,21,22,27,28,29,30,31], whereas 625 out of 784 reported an improvement of 1 or more [2,3,4,8,10,16,17,18,19,20,21,22,27,28,29,30,31,32]. A score of 4–5, recognized as postoperative recovery, was reported in 475 patients out of 918 [2,3,4,8,9,10,15,16,17,18,19,20,21,22,27,28,29,30,31,32]. The proportion estimates, confidence intervals, I^2^ values, included study *n* values, and patient sample size *n* values of MMT scoring have been summarized from forest plots in Table 5.

VAS scores were reported in five study groups, with a total sample size of 286 [9,16,17,18,19]. The preoperative VAS mean was 5.91 (95% CI: 4.21–7.60, I^2^ = 99%). The postoperative VAS mean was 1.00 (95% CI: −0.05–2.06, I^2^ = 99%).

Complications were documented in seven studies, with a total sample size of 301. There were no reports in most studies [17,18,19,21,28], except Girardi, Cammisa [2], who reported one patient with wound seroma, and Wang, Zhang [9], who reported two patients with intraoperative dural laceration and postoperative paraesthesia. The overall complications proportion across studies was 0.01 (95% CI: −0.00–0.02, I^2^ = 0%) [2,9,17,18,19,21,28]. Re-operations were reported in three studies, with a total of seven reports from a sample size of one hundred twenty-seven [2,9,28]. The re-operation proportion was 0.04 (95% CI: 0.00–0.09, I^2^ = 32%) [2,9,28]. The forest plots for postoperative outcomes are available as Supplementary Figures S8–S11. Visual inspection of all postoperative MMT outcome funnel plots indicated relative symmetry. For VAS and complication outcomes, the inclusion of fewer than 10 studies limited the distinguishing chance from real asymmetry. Funnel plots are available in Supplementary Figure S12.

## 4. Discussion

Whilst spinal decompression for foot drop arising from LDD remains the treatment of choice for suitable surgical patients, there exists room for a deeper understanding of responders and non-responders. Our meta-analysis demonstrates that up to 82% of patients will see some form of improvement in their postoperative MMT scoring, with up to 60% demonstrating clinically classified recovery. Up to 24% of patients are expected to require fusion during operation; however, overall complication rates are minimal at 1%, with re-operation requirements also low at 4%. Pain scores, as measured by VAS, are also seen to improve to near baseline levels. Despite further evidence of surgical intervention success, the findings here highlight a subset of patients who do not experience complete recovery, with 18% demonstrating no improvement at all. Whilst all patients suitable for surgery should be offered intervention, the results of this meta-analysis encapsulate the current literature state to provide a stepwise evidence-based framework for elucidating refractory cohorts.

Overall, our meta-analysis provides an up-to-date corroboration of the current cohort literature to demonstrate that LDD is highly effective in treating foot drop according to postoperative MMT scoring. The results here align with those of Saeed, Mukherjee [11], in which nine included studies of 431 patients demonstrated an MMT improvement rate of 84.5%. With over double the patient size from the recency of included studies, our findings are similar at 82%. However, the novelty within the current meta-analysis is the explicit elucidation of failure rates at 18%, giving a clinical perspective of higher refractory rates to surgical intervention than what the previous literature has dictated.

Factors surrounding the success of surgical intervention require continued exploration. Saeed, Mukherjee [11] investigated prognostic factors within their study, finding the duration of symptoms, preoperative MMT severity, and age to be factors for consideration. However, the marring of results due to the low sample sizes of the included studies was more suggestive of future direction than definitive evidence. The authors proposed this could be addressed through a national and international database, which has yet to be implemented [11]. Another meta-analysis using six retrospective studies by Song, Nam [1] found that decompression within one month was more beneficial than late surgery after the evaluation of neurological function and recovery rate postoperatively. Despite this, the timing of surgery has shown inconsistent effects [4,28]. Considering the sample size of 157 early decompression against 155 late decompression patients, the authors acknowledged the need for higher-quality clinical trials and RCTs to support their findings.

Baig Mirza, Khizar Khoja [12] are the most recent authors to attempt an investigation of successful prognostic factors through the preliminary prompts of Saeed, Mukherjee [11], and Song, Nam [1]. Successful factors of younger age, shorter duration of symptoms, higher preoperative MMT, and improved postoperative MMT within 3 months of surgery were identified [12]. However, to arrive at this conclusion, Baig Mirza, Khizar Khoja [12] required the utilization of case reports through an individualised dataset of 66 records with disc herniation only, citing a paucity of robust evidence reported within the literature to elaborate any further. Worth remarking is that baseline MMT severity, and correlated improvement is, at present, rarely delineated well. This posed a substantial source of heterogeneity in our postoperative outcomes because of pooled grouping.

Another meta-analysis by Hou, Liang [34] identified existing comorbidities as possible confounding for surgical outcomes. Through their study, it was demonstrated that diabetes mellitus had a poorer prognosis. However, only two included studies were a part of their analysis. Baig Mirza, Khizar Khoja [12] also attempted further elaboration of comorbidities but were unable to pool their individualised datasets. On a cohort level, the findings of the current meta-analysis shared the same sentiments, precluding sub-group analysis. This was compounded by a relatively low percentage of included populations having reported existing conditions and impairments outside of gluteal medius paralysis [2,3,8,10,19,20,21,22,27,28,29,30,31] and radiculopathy [2,3,8,10,19,20,21,22,27,28,29,30,31].

The conclusions drawn from prior meta-analyses and the findings here of cohort data highlight the current landscape of the medical literature regarding surgically treated foot drop. Despite the prompts of the duration of symptoms, preoperative MMT severity, age, improvement within 3 months, and comorbidities, such as diabetes mellitus as prognostic factors [1,11,12,34], reporting in published articles remains poor. This, in turn, prevented any sub-group analysis that could have been conducted by the current meta-analysis to further the field forward in this aspect.

Evidence on the conservative management of foot drop is additionally scarce [35], with daily physiotherapy recommended in the context of peroneal nerve palsy [36]. While previous studies have found that surgical intervention for LSS has better long-term outcomes compared with conservative management [37], there is very little data available in the context of LDD-caused foot drop. The omission of any prior conservative treatment within the reporting of included articles or direct comparisons to surgery in the literature impeded any investigation within the current meta-analysis. While it is currently accepted practice to attempt conservative management prior to surgical intervention in mechanical spinal pathologies [38], there is minimal evidence to confirm whether this improves long-term outcomes or if surgical management should be the first choice intervention for foot drop.

There is also no standardized follow-up window for patients who undergo surgery for foot drop; the mean follow-up range reported in our analysis ranges widely from 0.03 to 81.00 months. As a result of this, long-term postoperative outcomes remain a critical point of investigation. Previous publications have shown that some nerve fibers take up to one year to heal [39,40]. For those studies with shorter follow-up periods, it is possible postoperative MMT scores may have improved beyond the reported scores. Standardized and increased follow-up periods in future studies could allow for an improved understanding of long-term symptom progression and function postoperatively. The high variance in follow-up of included studies could have also contributed to the heterogeneity seen within this analysis.

Of unique observation within the current meta-analysis, discectomy appeared as the surgical modality of choice, comprising nearly half of procedures (20% microdiscectomy, 19% open discectomy, and 10% transforaminal discectomy). Given that 79% of patients had disc herniation, mostly attributed to a disc protrusion, it is understandable that the surgical intervention choice reflected this. Therefore, the current meta-analysis presents evidence of foot drop as a neurological sequelae of disc herniation in concordance with the literature [41]. In treating disc herniation, discectomy has been proven to be the definitive treatment modality in both adults [42] and children [33], which the current results reflect due to the increased MMT scores postoperatively. Complementary to this, 22% of patients were reported to have spinal stenosis, with 6% having undergone laminectomy or laminotomy and 14% having undergone unspecified decompression. In all instances, the end goal is to relieve compression of the affected neurological structures, resulting in the alleviation of any symptomology [43]. These findings pose questions on which subsection of LDD is more attributable to foot drop. It could be that herniation is simply a more common etiology over stenosis in foot drop pathologies, or it could be that herniation is more likely to result in symptomology than stenosis. Future studies investigating these factors, as well as the severity of herniation or stenosis on the likelihood of foot drop presentation, require elaboration. This remains true for the affected lumbar level, as a rarity in correlated reporting of postoperative MMT did not allow for meta-analysis. The wide range of surgical modalities could have also contributed to the high heterogeneity seen in our postoperative outcomes and pain scoring. Similarly, Vande Kerckhove, d’Astorg [44] commented on differences in surgical technique causing heterogeneity within their meta-analysis of lumbar foraminal stenosis. Arguments thus arise for future studies to pool select surgical modalities in a bid to mitigate heterogeneity and strengthen conclusions.

Considering the context of the prior literature and the findings here, the value of this meta-analysis is the attention to ongoing knowledge gaps surrounding spinal decompression for LDD-caused foot drop. By acknowledging this dearth of evidence, there are compelling arguments for further exploration through targeted study design and purposeful reporting. Without such evidence-based literature studies, floating expert opinion will prevail without a lack of standardized guidelines to optimize patient outcomes in accordance with validated prognostic factors.

## 5. Limitations

As with all meta-analyses, limitations are present. Foremost is the paucity of clear reporting within included articles, as stated previously. Stratification of datasets could not be achieved to investigate factors such as duration of symptoms, partial or complete recovery postoperatively, preoperative MMT severity, age, improvement within 3 months, existing comorbidities, herniation grading or canal stenosis severity, and surgical modality on outcomes. This highlights the need for a clear delineation of datasets to enable such analyses. Second, the high heterogeneity seen in the current outcomes dictates a reserved interpretation of results. This heterogeneity could be in relation to the relatively small sample size, diversity in patient populations, surgical methodology, and follow-up periods within the current dataset. Patient demographics, in particular, are stated by Issa, Lambrechts [45] to be poorly reported within the spine literature, preventing stratification. This may restrict the ability to generalize these results to a larger population. Third, the lack of RCTs within the literature prevented any form of two-arm analysis to validate our results further. This is especially prevalent in relation to conservative measures, surgical modalities, or combined postoperative rehabilitation regimes. Compounded by the predominantly retrospective design of the included studies, patient-selection bias remains a drawback of the current meta-analysis. However, this is, by and large, a reflection of the existing state of the literature, hence the nature of the overall quality of evidence found within this meta-analysis. Fourth, a lack of reporting on failed preoperative conservative measures prohibited any insight into the management employed prior to the requirement for surgery. This may include physical therapy, foot orthotics, and nerve stimulation modalities. Fifth, for ethical reasons, patients could not be randomized to further supplemental treatments such as analgesics or undocumented therapies, and a lack of adequate reporting prevented controlling for their effects. Last, the current meta-analysis could not control for surgeon experience and elucidate any effect this may have had on postoperative outcomes.

## 6. Conclusions

The current meta-analysis is the most comprehensive and up-to-date corroboration of the existing literature to characterize spinal decompression for foot drop caused by LDD. Where indicated, lumbar decompression remains the ideal treatment modality for improving MMT scoring and alleviating symptoms in up to 82% of patients. However, the results here show that up to 18% of patients will not respond to surgery, commanding attention to this cohort. As such, although the treatment of choice for suitable candidates, surgical decompression may not be a one-size-fits-all solution with the need for further investigations. By encapsulating the current literature state, this meta-analysis gives direction for future randomized or prospective studies, with a clear delineation of datasets, to extrapolate further prognostic factors that may elucidate those unlikely to respond to foot drop treated with lumbar decompression.

## Figures and Tables

**Table 1:** Study characteristics.

Study	Country	Study Design	Patient (*n*)	M:F	Mean Age (Years) ± SD	Mean Follow-Up (Months) ± SD	Pain Status
Albayrak et al. (2016) [32]	Turkey	Retrospective	42	30:2	46.70 ± 9.93	17.80 ± 14.55	NR
Aono et al. (2014) [10]	Japan	Retrospective	20	12:8	52.00 ± 14.25	46.80 ± 21.00	Painless
Aono et al. (2007) [27]	Japan	Retrospective	46	27:19	56.60 ± 15.75	44.43 ± 21.02	Both
Berger et al. (2022) [28]	Israel	Retrospective	40	24:16	58.90 ± 17.90	62.40 ± 27.60	Pain
Bhargava et al. (2012) [8]	UK	Retrospective	26	15:11	48.00 ± 13.50	NR	Pain
Demetriades et al. (2021) [21]	UK	Case series	7	6:1	52.10 ± 6.25	20.86 ± 27.75	Pain
Galal (2013) [29]	Egypt	Retrospective	31	14:17	NR	9.80 ± NR	Both
Gao et al. (2023) [16]	China	Retrospective	87	52:35	54.00 ± 11.00	81.00 ± 24.00	Pain
Girardi et al. (2002) [2]	USA	Retrospective	55	40:15	46.80 ± 13.00	36.00 ± 31.00	Pain
Iizuka et al. (2009) [30]	Japan	Retrospective	28	16:12	55.00 ± 12.75	19.80 ± 13.50	NR
Kumar et al. (2021) [22]	India	Case series	3	3:0	62.00 ± 7.75	1.33 ± 0.25	Painless
Liu et al. (2013) [17]	China	Retrospective	135	62:73	55.00 ± 5.25	27.60 ± 3.00	Pain
Masuda et al. (2020) [20]	Japan	Retrospective	87	52:35	66. 90 ± 14.75	20.30 ± 16.50	Both
Nakashima et al. (2020) [4]	Japan	Retrospective	60	38:22	54.60 ± 14.25	43.20 ± 10.20	NR
Sun et al. (2021) [18]	China	Prospective	27	21:6	46.00 ± 10.75	19.00 ± 3.75	Pain
Takenaka & Aono (2017) [15]	Japan	Retrospective	102	58:44	59.00 ± 17.03	30.00 ± 13.33	Both
Tanaka et al. (2021) [3]	Japan	Retrospective	55	37:18	61.80 ± 16.25	20.30 ± 11.25	Both
Telfeian et al. (2019) [19]	USA	Case series	5	1:4	65.60 ± 8.25	12.00 ± 0.00	Pain
Wang et al. (2017) [9]	China	Retrospective	32	18:14	48.70 ± 8.60	24.00 ± 0.00	Pain
Yüksel et al. (2019) [31]	Turkey	Retrospective	30	12:18	46.50 ± 13.50	0.03 ± 0.00	NR

Abbreviations. F: Female; M: Male; NR: Not reported; SD: Standard deviation.

**Table 2:** Preoperative characteristics.

	Proportion	95% CI	I^2^	Included Study Groups (*n*)	Sample Size (*n*)
Operative level					
L1/L2	<0.01	<0.01–0.01	0%	17	749
L2/L3	0.02	<0.01–0.02	16%	17	749
L3/L4	0.20	0.12–0.60	89%	17	749
L4/L5	0.79	0.72–0.86	87%	17	749
L5/S1	0.25	0.16–0.33	90%	17	749
Involved Levels					
Single-level	0.73	0.63–0.83	96%	20	918
Multi-level	0.27	0.17–0.37	96%	20	
Aetiology					
Disc herniation	0.79	0.72–0.85	96%	19	891
Spinal stenosis	0.22	0.15–0.30	96%	19	891
Spondylolisthesis	0.02	0.01–0.03	31%	19	891
Spondylolysis	0.01	<0.01–0.01	0%	19	891
Spondylitis	0.01	<0.01–0.01	0%	19	891
Herniation Grading					
Bulging	0.02	<0.01–0.05	0%	7	157
Protrusion/prolapse	0.47	0.04–0.89	100%	7	157
Extrusion	0.19	0.06–0.32	91%	7	157
Sequestration	0.24	0.09–0.39	94%	7	157
Foot Drop Laterality					
Unilateral	0.87	0.83–0.94	92%	15	706
Bilateral	0.11	0.06–0.17	92%	15	706
Preoperative Imaging					
Radiography	0.26	−0.05–0.57	100%	9	227
CT	0.16	−0.09–0.40	100%	9	227
MRI	0.99	0.97–1.00	0%	10	269

Abbreviations. CI: Confidence interval; CT: Computed tomography; MRI: Magnetic resonance imaging.

**Table 3:** Primary surgical modality.

	Proportion	95% CI	I^2^	Included Study Groups (*n*)	Sample Size (*n*)
Primary Surgical Modality					
Fenestration	0.04	0.02–0.05	82%	20	918
Laminectomy/Laminotomy	0.06	0.03–0.08	92%	20	918
Microdiscectomy	0.20	0.10–0.30	100%	20	918
Open Discectomy	0.19	0.20–0.24	96%	20	918
Spondylolysis Repair	0.01	<0.01–0.01	0	20	918
Transforaminal Endoscopic Discectomy	0.10	0.04–0.16	99%	20	918
Tubular Discectomy	0.01	<0.01–0.03	66%	20	918
Unspecified Decompression	0.14	−0.08–0.36	100%	20	918
Fusion	0.24	0.01–0.46	100%	20	918

Abbreviations. CI: Confidence interval.

**Table 4:** Comorbidities and impairments.

	Proportion	95% CI	I^2^	Included Study Groups (*n*)	Sample Size (*n*)
Comorbidities					
Cauda Equina	0.04	0.01–0.06	75%	16	637
Diabetes	0.04	0.02–0.06	64%	16	637
Dyslipidaemia	0.01	<0.01–0.02	0%	16	637
Hypertension	0.02	0.01–0.04	47%	16	637
Ischemic Heart Disease	0.01	<0.01–0.02	0%	16	637
Osteoporosis	0.01	<0.01–0.02	0%	16	637
Spinal Trauma	0.01	<0.01–0.02	0%	16	637
Other *	0.03	0.01–0.04	65%	16	637
Impairments					
Gluteus Medius Paralysis	0.15	−0.06–0.37	100%	16	637
Radiculopathy	0.48	0.24–0.72	100%	16	637

* Other classified as smokers *n* = 23, rheumatoid arthritis *n* = 6, COPD *n* = 3, and asthma *n* = 1. Abbreviations. CI: Confidence interval; COPD: Chronic obstructive pulmonary disease.

**Table 5:** Manual muscle test scores.

	Proportion	95% CI	I^2^	Included Study Groups (*n*)	Sample Size (*n*)
Preoperative MMT					
0–1	0.47	0.24–0.70	99%	16	764
2–3	0.51	0.34–0.68	97%	16	764
4	0.01	<0.01–0.02	44%	16	764
Postoperative MMT					
0–1	0.13	0.08–0.18	88%	15	650
2–3	0.24	0.13–0.36	95%	15	650
4–5 (recovery)	0.60	0.44–0.75	97%	20	918
Increase of MMT ≥ 1	0.82	0.76–0.88	89%	18	784
No improvement *	0.18	0.12–0.24	89%	17	724

* No improvement was defined by included study authors as no change between pre and postoperative MMT. Abbreviations. CI: Confidence interval, MMT: Manual muscle testing.

## Data Availability

The dataset used for this meta-analysis will be shared upon request from the study authors.

## Supplementary Materials

The following supporting information can be downloaded at: https://www.mdpi.com/article/10.3390/jcm14134470/s1, Supplemental Item S1: Actual search strategies. Figure S1: Affected levels. Figure S2: Foot drop characteristics. Figure S3: Preoperative imaging. Figure S4: Etiology. Figure S5: Herniation grading. Figure S6: Surgical modalities. Figure S7: Comorbidities and impairments. Figure S8: Preoperative manual muscle test (MMT) scoring. Figure S9: Postoperative manual muscle test (MMT) scoring. Figure S10: Visual analogue scale (VAS). Figure S11: Complications and re-operation. Figure S12: Funnel plots.

## Abbreviations

The following abbreviations are used in this manuscript:

CI	Confidence Interval
CT	Computed Tomography
LDD	Lumbar Degenerative Diseases
LDH	Lumbar Disc Herniation
LSS	Lumbar Spinal Stenosis
MRC	Medical Research Council
MMT	Manual Muscle Test
MRI	Magnetic Resonance Imaging
PRISMA	Preferred Reporting Items for Systematic reviews and Meta-Analyses
RCT	Randomized Controlled Trials
ROBINS-I	Risk Of Bias In Non-randomized Studies-of Interventions
SD	Standard Deviation
VAS	Visual Analogue Scale
