# Supplementary material for: Characterizing Spinal Decompression for Foot Drop Caused by Lumbar Degenerative Disease: A Systematic Review and Meta-Analysis of Cohorts
Source: J Clin Med. 2025 Jun 24;14(13):4470. doi: 10.3390/jcm14134470 (PMC12249779; doi:10.3390/jcm14134470)
Supplement: Supplementary file 1 [file jcm-14-04470-s001.zip › jcm-3690927-supplementary.pdf]

Supplemental Material

Supplemental Item S1: Actual Search Strategies

PubMed

Foot drop OR "dangle foot" OR "drop foot" OR "dropfoot" OR "dropping foot" OR "foot drop" OR "footdrop" OR "peroneal nerve palsy" OR "peroneal nerve paralysis" OR "peroneal palsy" OR peroneus palsy

5116

AND

disc herniation OR "disc hernia" OR "disc herniation" OR "disc prolapse" OR "discal hernia" OR "discal herniation" OR "discus hernia" OR "disk hernia" OR "disk herniation" OR "disk prolapse" OR "disk protrusion" OR "disk, herniated" OR "hernia nuclei pulposi" OR "herniated disc" OR "herniated disk" OR "herniated intervertebral disc" OR "herniated intervertebral disk" OR "herniated nucleus pulposus" OR "herniated vertebral disc" OR "herniated vertebral disk" OR "intervertebral disc displacement" OR "intervertebral disc hernia" OR "intervertebral disc prolapse" OR "intervertebral disc protrusion" OR "intervertebral disk displacement" OR "intervertebral disk herniation" OR "intervertebral disk prolapse" OR "intervertebral disk rupture" OR "intervertebral prolapse" OR "nucleus pulposus hernia" OR "slipped disc" OR "slipped disk" OR "slipped intervertebral disc" OR "spinal disc prolapse" OR "spinal disk disease" OR "vertebral disk hernia" OR "intervertebral disk hernia" OR spinal stenosis OR "spinal canal stenosis" OR "spinal stenosis" OR "vertebral canal stenosis" OR degenerative lumbar disease

44793

AND

Discectomy OR Lumbar Fusion OR Spine Surgery OR Lumbar decompression OR spinal surgery OR intervertebral disk removal OR intervertebral disc removal OR Diskectomy

254173

146

Since 10/6/24 6*

Embase

No.	Query Results	Results	Date
#22.	#3 AND #7 AND #20 AND [10-06-2024]/sd NOT [01-05-2025]/sd	7	21 Mar 2025
#21.	#3 AND #7 AND #20	173	21 Mar 2025
#20.	#8 OR #9 OR #10 OR #11 OR #12 OR #13 OR #14 OR #15 OR #16 OR #17 OR #18 OR #19	272,506	21 Mar 2025
#19.	'intervertebral disc removal'	10	21 Mar 2025
#18.	'intervertebral disk removal'	3	21 Mar 2025
#17.	'spinal surgery'	18,378	21 Mar 2025
#16.	spinal AND surgery	205,006	21 Mar 2025
#15.	'lumbar decompression'	1,523	21 Mar 2025
#14.	'lumbar decompression'/exp	55	21 Mar 2025
#13.	'spinal surgery' OR 'spinal vertebral surgery' OR 'spine surgery'	69,110	21 Mar 2025
#12.	'spine surgery'/exp	124,558	21 Mar 2025
#11.	'lumbar fusion' OR 'lumbar fusion surgery' OR 'lumbar spinal fusion' OR 'lumbar spine fusion' OR 'lumbar vertebra fusion' OR 'lumbar vertebrae fusion' OR 'lumbar vertebral fusion' OR 'lumbar interbody fusion'	14,054	21 Mar 2025
#10.	'lumbar interbody fusion'/exp	5,792	21 Mar 2025
#9.	'diskectomy' OR 'diskectomy, intervertebral' OR 'intervertebral disc removal' OR 'intervertebral discectomy' OR 'intervertebral disk removal' OR 'intervertebral diskectomy' OR 'discectomy'	19,589	21 Mar 2025
#8.	'discectomy'/exp	17,743	21 Mar 2025
#7.	#4 OR #5 OR #6	36,131	21 Mar 2025
#6.	'degenerative lumbar disease'/exp	45	21 Mar 2025
#5.	'disc hernia' OR 'disc herniation' OR 'disc prolapse' OR 'discal hernia' OR 'discal herniation' OR 'discus hernia' OR 'disk hernia' OR 'disk hernia disease' OR 'disk herniation' OR 'disk prolapse' OR 'disk protrusion' OR 'disk, herniated' OR 'hernia disci' OR 'hernia nuclei pulposi' OR 'herniated disc' OR 'herniated disk' OR 'herniated intervertebral disc' OR 'herniated intervertebral disk' OR 'herniated nucleus pulposus' OR 'herniated vertebral disc' OR 'herniated vertebral disk' OR 'intervertebral disc displacement' OR 'intervertebral disc hernia' OR 'intervertebral disc prolapse' OR 'intervertebral disc protrusion' OR 'intervertebral disk displacement' OR 'intervertebral disk herniation' OR 'intervertebral disk perforation' OR 'intervertebral disk prolapse' OR 'intervertebral disk protrusion' OR 'intervertebral disk rupture' OR 'intervertebral prolapse' OR	36,095	21 Mar 2025

'invertebral disk hernia' OR 'nucleus pulposus hernia' OR 'rupture, intervertebral disk' OR 'slipped disc' OR 'slipped disk' OR 'slipped intervertebral disc' OR 'slipped vertebral disc' OR 'spinal disc prolapse' OR 'spinal disk disease' OR 'spinal disk hernia' OR 'vertebral disc hernia' OR 'vertebral disk hernia' OR 'intervertebral disk hernia'
#4. 'intervertebral disk hernia'/exp 32,999 21 Mar 2025
#3. #1 OR #2 6,498 21 Mar 2025
#2. 'dangle foot' OR 'drop foot' OR 'dropfoot' OR 'dropping foot' OR 'foot drop' OR 'footdrop' OR 'palsy, peroneus' OR 'paralysis, peroneal nerve' OR 'peroneal nerve palsy' OR 'peroneal nerve paralysis' OR 'peroneal palsy' OR 'peroneus palsy' OR 'peroneus nerve paralysis' 6,498 21 Mar 2025
#1. 'peroneus nerve paralysis'/exp 4,199 21 Mar 2025

Cochrane
Search Name:
Date Run: 21/03/2025 05:18:18
Comment:
ID Search Hits
#1 MeSH descriptor: [Peroneal Neuropathies] explode all trees 32
#2 (Foot drop OR "dangle foot" OR "drop foot" OR "dropfoot" OR "dropping foot" OR "foot drop" OR "footdrop" OR "peroneal nerve palsy" OR "peroneal nerve paralysis" OR "peroneal palsy" OR peroneus palsy):ti,ab,kw (Word variations have been searched) 837
#3 #1 OR #2 846
#4 MeSH descriptor: [Intervertebral Disc Displacement] explode all trees 1381
#5 MeSH descriptor: [Spinal Stenosis] explode all trees 682
#6 (disc herniation OR "disc hernia" OR "disc herniation" OR "disc prolapse" OR "discal hernia" OR "discal herniation" OR "discus hernia" OR "disk hernia" OR "disk herniation" OR "disk prolapse" OR "disk protrusion" OR "disk, herniated" OR "hernia nuclei pulposi" OR "herniated disc" OR "herniated disk" OR "herniated intervertebral disc" OR "herniated intervertebral disk" OR "herniated nucleus pulposus" OR "herniated vertebral disc" OR "herniated vertebral disk" OR "intervertebral disc displacement" OR "intervertebral disc hernia" OR "intervertebral disc prolapse" OR "intervertebral disc protrusion" OR "intervertebral disk displacement" OR "intervertebral disk herniation" OR "intervertebral disk prolapse" OR "intervertebral disk rupture" OR "intervertebral prolapse" OR "nucleus pulposus hernia" OR "slipped disc" OR "slipped disk" OR "slipped intervertebral disc" OR "spinal disc prolapse" OR "spinal disk disease" OR "vertebral disk hernia" OR "intervertebral disk hernia" OR spinal stenosis OR "spinal canal stenosis" OR "spinal stenosis" OR "vertebral canal stenosis" OR degenerative lumbar disease):ti,ab,kw (Word variations have been searched) 5911
#7 #4 OR #5 OR #6 5911
#8 MeSH descriptor: [Diskectomy] explode all trees 780
#9 (Discectomy OR Lumbar Fusion OR Spine Surgery OR Lumbar decompression OR spinal surgery OR intervertebral disk removal OR intervertebral disc removal OR Diskectomy):ti,ab,kw (Word variations have been searched) 22447
#10 #8 OR #9 22447
#11 #3 AND #7 AND #10 5 No new items

Scopus
(TITLE-ABS-KEY ("dangle foot" OR "drop foot" OR "dropfoot" OR "dropping foot" OR "foot drop" OR "footdrop" OR "peroneal nerve palsy" OR "peroneal nerve paralysis" OR "peroneal palsy" OR "peroneus palsy")) AND (TITLE-ABS-KEY ("disc hernia" OR "disc herniation" OR "disc prolapse" OR "discal hernia" OR "discal herniation" OR "discus hernia" OR "disk hernia" OR "disk herniation" OR "disk prolapse" OR "disk protrusion" OR "disk, herniated" OR "hernia nuclei pulposi" OR "herniated disc" OR "herniated disk" OR "herniated intervertebral disc" OR "herniated intervertebral disk" OR "herniated nucleus pulposus" OR "herniated vertebral disc" OR "herniated vertebral disk" OR "intervertebral disc displacement" OR "intervertebral disc hernia" OR "intervertebral disc prolapse" OR "intervertebral disc protrusion" OR "intervertebral disk displacement" OR "intervertebral disk herniation" OR "intervertebral disk prolapse" OR "intervertebral disk rupture" OR "intervertebral prolapse" OR "nucleus pulposus hernia" OR "slipped disc" OR "slipped disk" OR "slipped intervertebral disc" OR "spinal disc prolapse" OR "spinal disk disease" OR "vertebral disk hernia" OR "intervertebral disk hernia" OR spinal AND stenosis OR "spinal canal stenosis" OR "spinal stenosis" OR "vertebral canal stenosis" OR "degenerative lumbar disease")) AND (TITLE-ABS-KEY (discectomy OR "Lumbar Fusion" OR "Spine Surgery" OR "Lumbar decompression" OR "spinal surgery" OR "intervertebral disk removal" OR "intervertebral disc removal" OR diskectomy))
39
2024-2025- 2 documents

Web of Science
Database: Web of Science Core Collection
Entitlements:
- WOS.IC: 1993 to 2025
- WOS.CCR: 1985 to 2025
- WOS.SCI: 1900 to 2025
- WOS.AHCI: 1975 to 2025
- WOS.BHCI: 2005 to 2025
- WOS.BSCI: 2005 to 2025
- WOS.ESCI: 2005 to 2025
- WOS.ISTP: 1990 to 2025
- WOS.SSCI: 1900 to 2025
- WOS.ISSHP: 1990 to 2025
Searches:

1: TS=("dangle foot" OR "drop foot" OR "dropfoot" OR "dropping foot" OR "foot drop" OR "footdrop" OR "peroneal nerve palsy" OR "peroneal nerve paralysis" OR "peroneal palsy" OR "peroneus palsy") Date Run: Fri Mar 21 2025 15:04:18 GMT+1000 (Australian Eastern Standard Time) Results: 3110

2: TS=("disc hernia" OR "disc herniation" OR "disc prolapse" OR "discal hernia" OR "discal herniation" OR "discus hernia" OR "disk hernia" OR "disk herniation" OR "disk prolapse" OR "disk protrusion" OR "disk, herniated" OR "hernia nuclei pulposi" OR "herniated disc" OR "herniated disk" OR "herniated intervertebral disc" OR "herniated intervertebral disk" OR "herniated nucleus pulposus" OR "herniated vertebral disc" OR "herniated vertebral disk" OR "intervertebral disc displacement" OR "intervertebral disc hernia" OR "intervertebral disc prolapse" OR "intervertebral disc protrusion" OR "intervertebral disk displacement" OR "intervertebral disk herniation" OR "intervertebral disk prolapse" OR "intervertebral disk rupture" OR "intervertebral prolapse" OR "nucleus pulposus hernia" OR "slipped disc" OR "slipped disk" OR "slipped intervertebral disc" OR "spinal disc prolapse" OR "spinal disk disease" OR "vertebral disk hernia" OR "intervertebral disk hernia" OR spinal stenosis OR "spinal canal stenosis" OR "spinal stenosis" OR "vertebral canal stenosis" OR "degenerative lumbar disease") Date Run: Fri Mar 21 2025 15:05:29 GMT+1000 (Australian Eastern Standard Time) Results: 27286

3: TS=(Discectomy OR "Lumbar Fusion" OR "Spine Surgery" OR "Lumbar decompression" OR "spinal surgery" OR "intervertebral disk removal" OR "intervertebral disc removal" OR Diskectomy) Date Run: Fri Mar 21 2025 15:06:05 GMT+1000 (Australian Eastern Standard Time) Results: 37991

4: #1 AND #2 AND #3 Date Run: Fri Mar 21 2025 15:06:32 GMT+1000 (Australian Eastern Standard Time) Results: 51

5: #1 AND #2 AND #3 and 2024 or 2025 (Publication Years) Date Run: Fri Mar 21 2025 15:06:55 GMT+1000 (Australian Eastern Standard Time) Results: 5

A

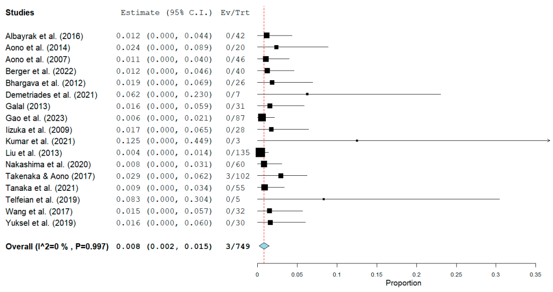

B

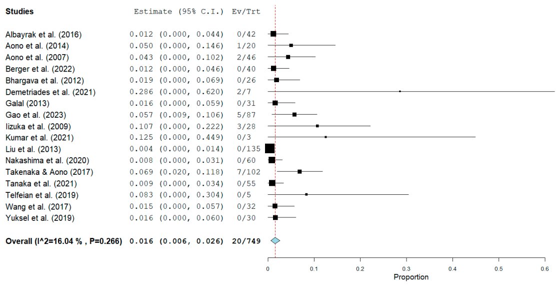

C

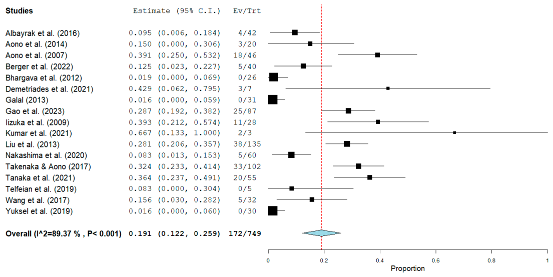

D

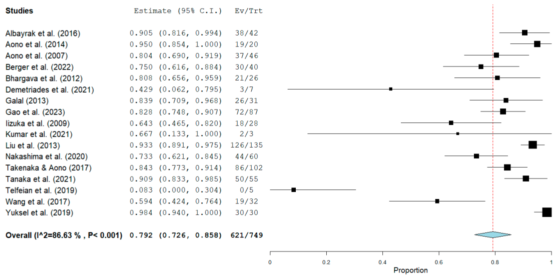

E

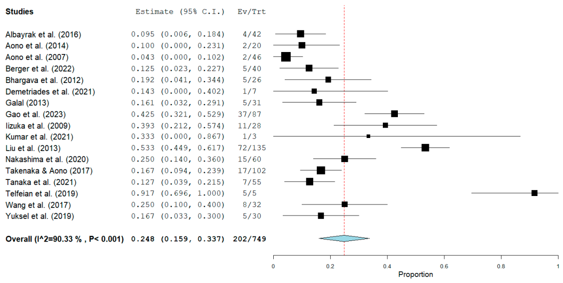

Supplementary Figure S1: Affected levels. A) L1/L2; B) L2/L3; C) L3/L4; D) L4/L5; E) L5/S1 (3, 4, 8-10, 15-17, 19, 21, 22, 27-32).

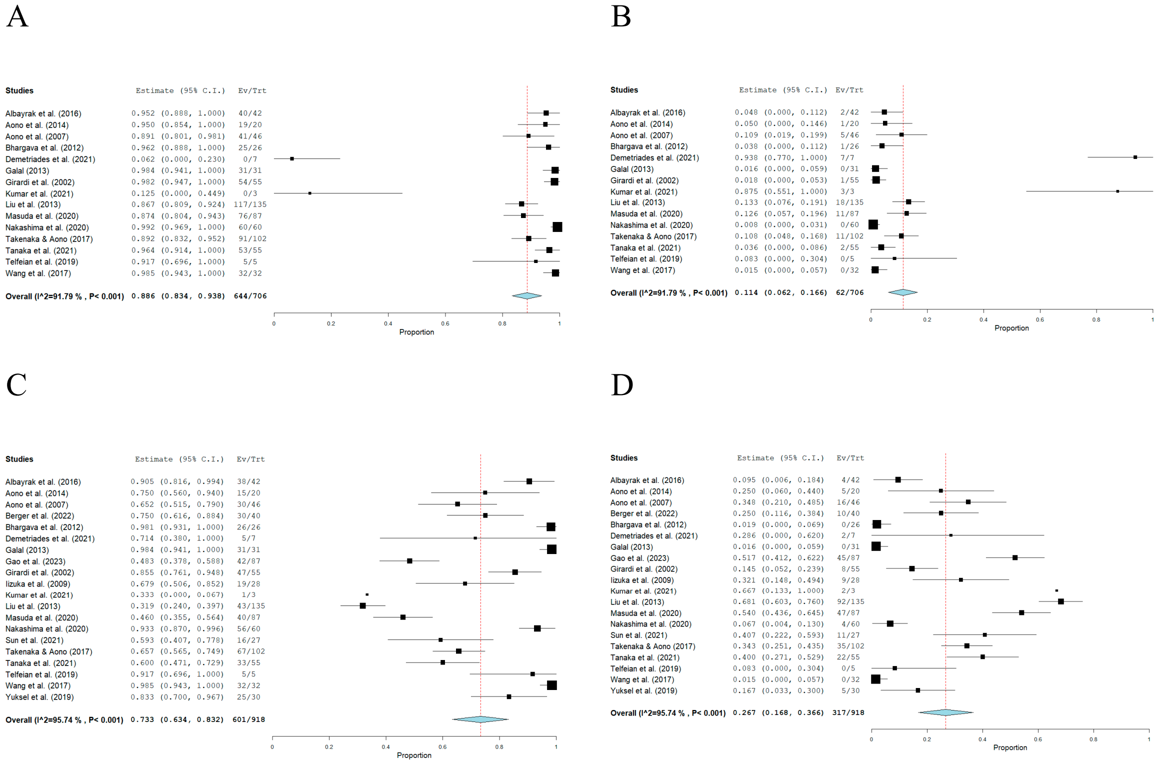

Supplementary Figure S2: Foot drop characteristics. A) Unilateral foot drop; B) Bilateral foot drop; C) Single level involvement; D) Multi-level involvement (2-4, 8-10, 15-22, 27-32).

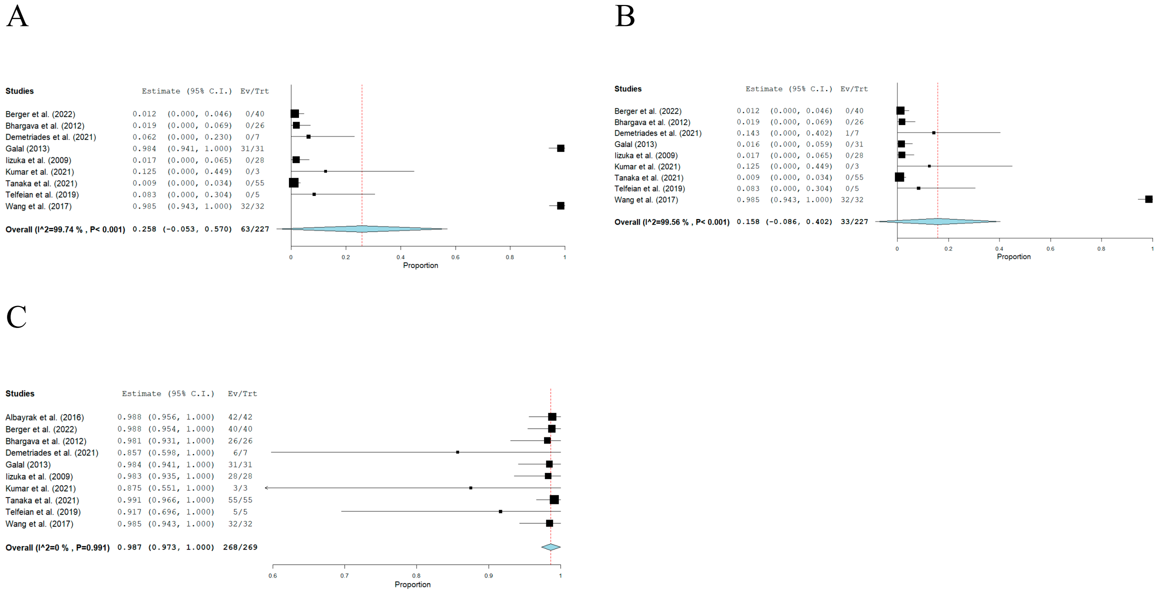

Supplementary Figure S3: Preoperative imaging. A) Radiography; B) Computed tomography (CT); C) Magnetic resonance imaging (MRI) (3, 8, 9, 19, 21, 22, 28-30, 32).

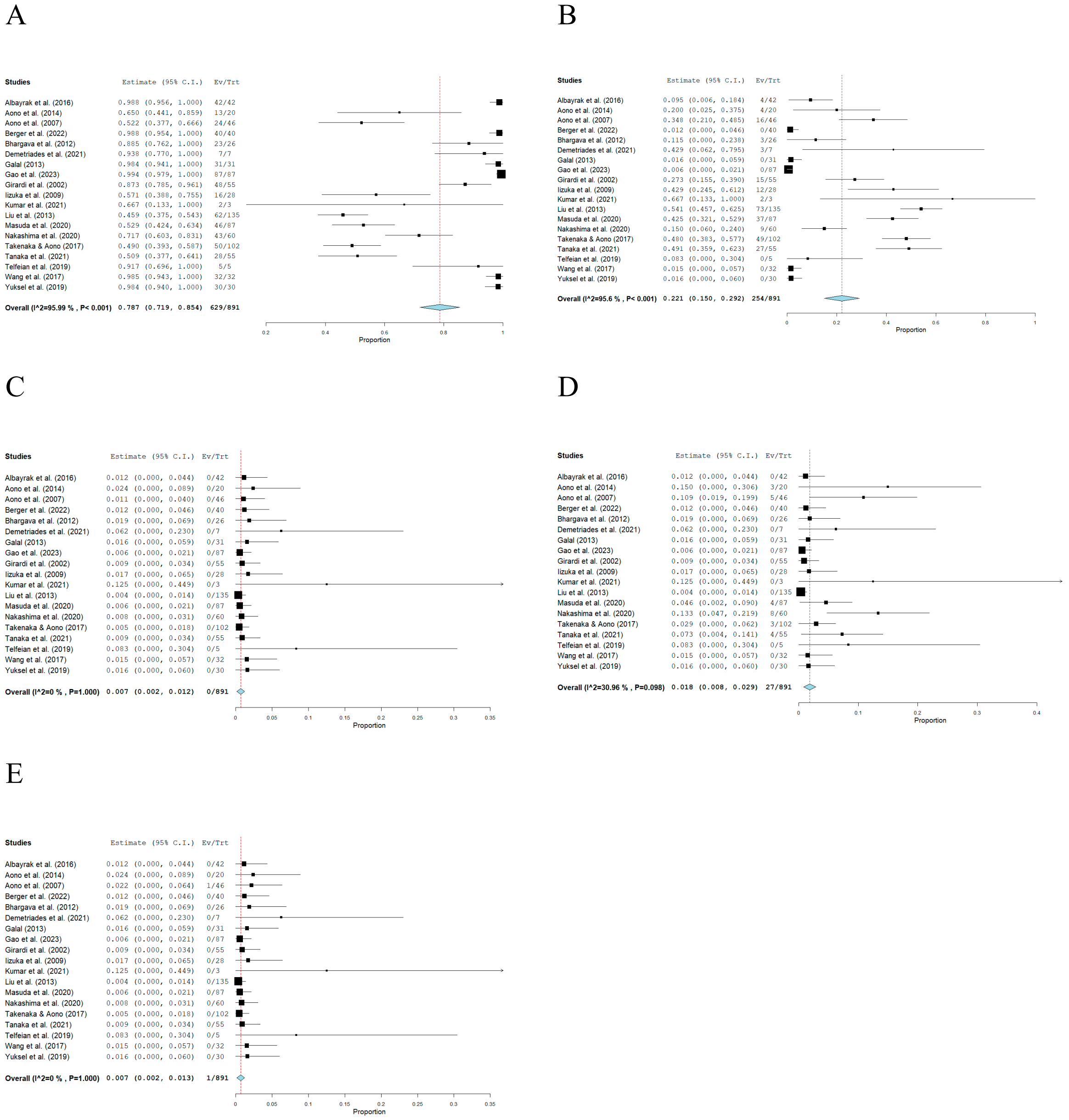

Supplementary Figure S4: Aetiology. A) Lumbar disc herniation; B) Lumbar spinal stenosis; C) Spondylitis; D) Spondylolisthesis; E) Spondylolysis (3, 4, 8-10, 15-17, 19-22, 27-32)

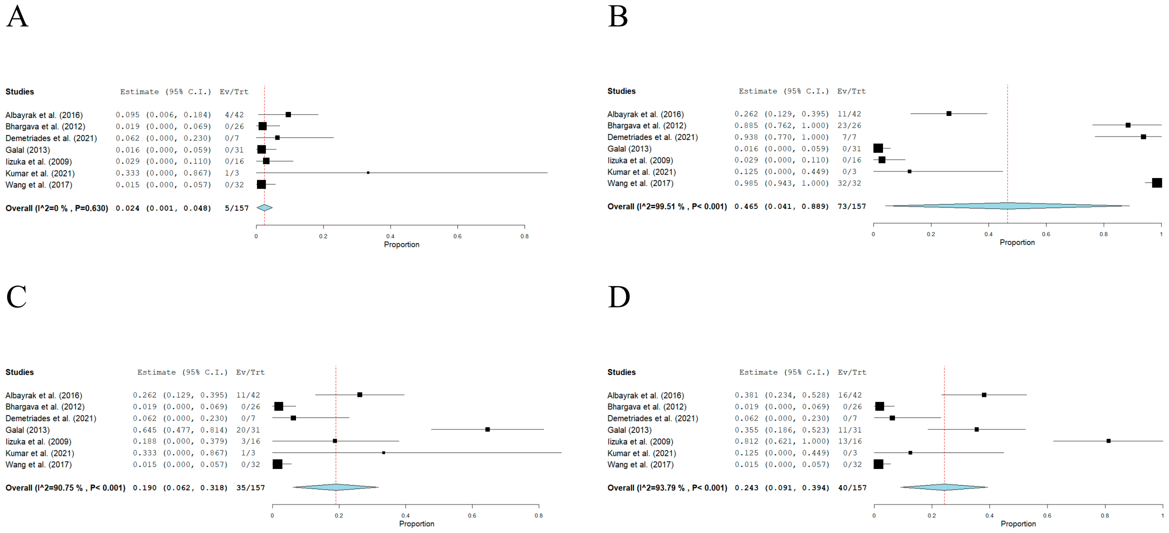

Supplementary Figure S5: Herniation grading. A) Bulge; B) Protrusion; C) Extrusion; D) Sequestration (2, 8, 9, 21, 22, 29, 30, 32).

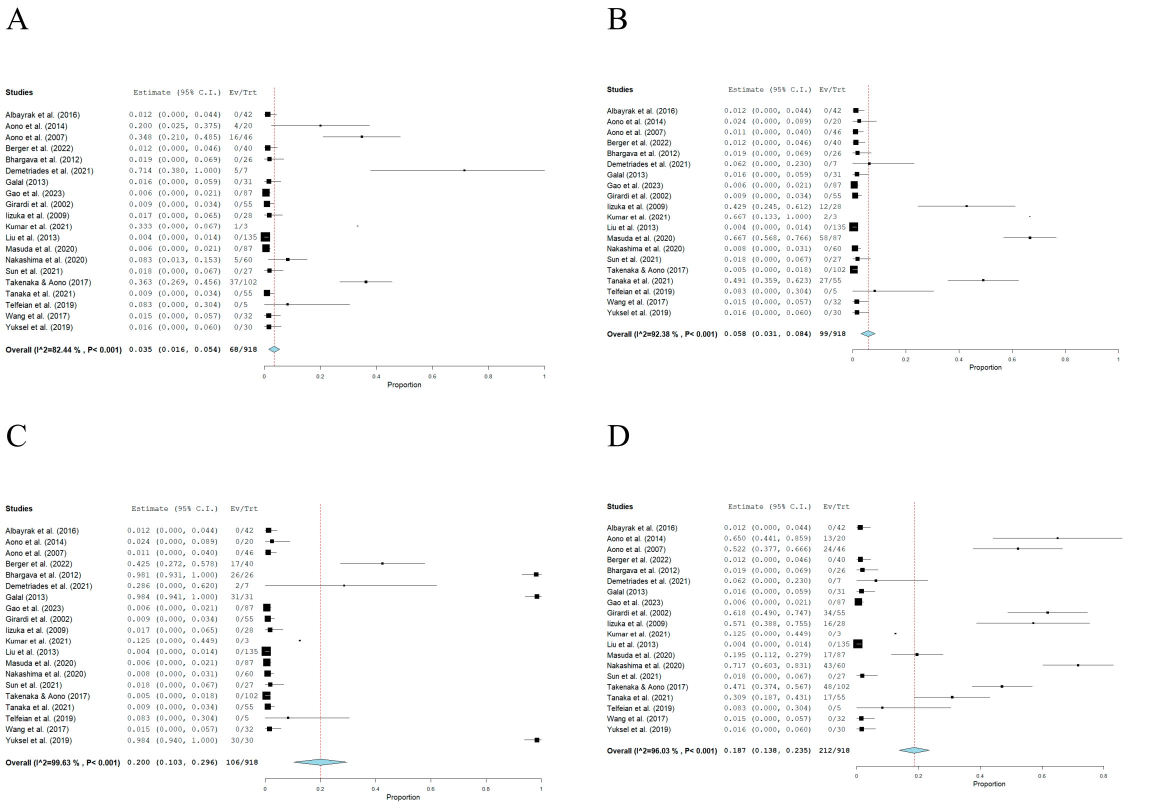

E

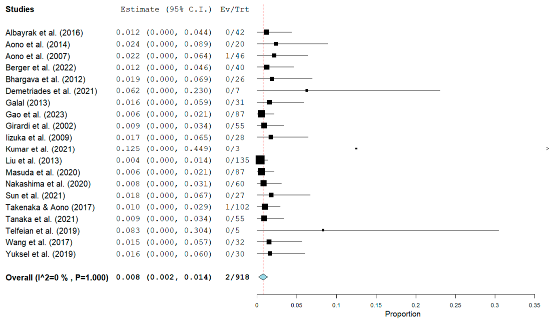

F

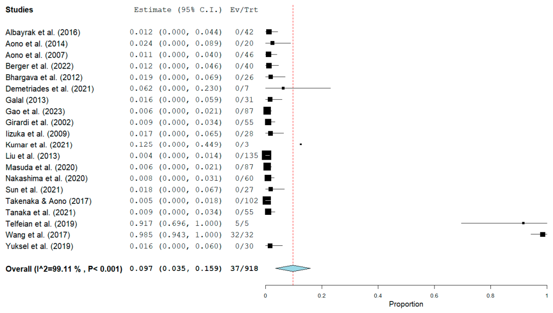

G

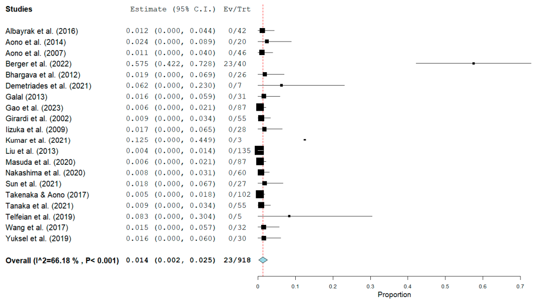

H

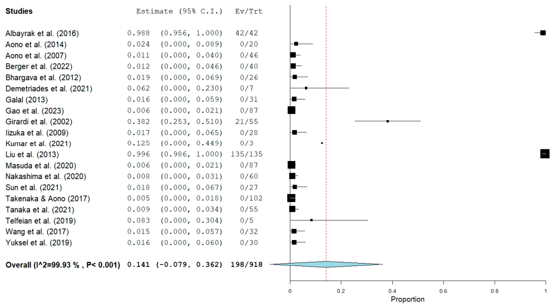

I

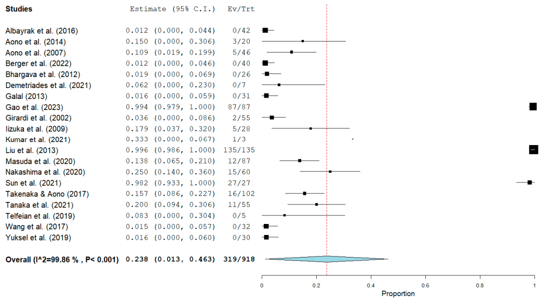

Supplementary Figure S6: Surgical modalities. A) Fenestration; B) Laminectomy/Laminotomy; C) Microdiscectomy; D) Open discectomy; E) Spondylolysis repair; F) Transforaminal endoscopic discectomy; G) Tubular discectomy; H) Unspecified discectomy; I) Fusion (2-4, 8-10, 15-22, 27-32).

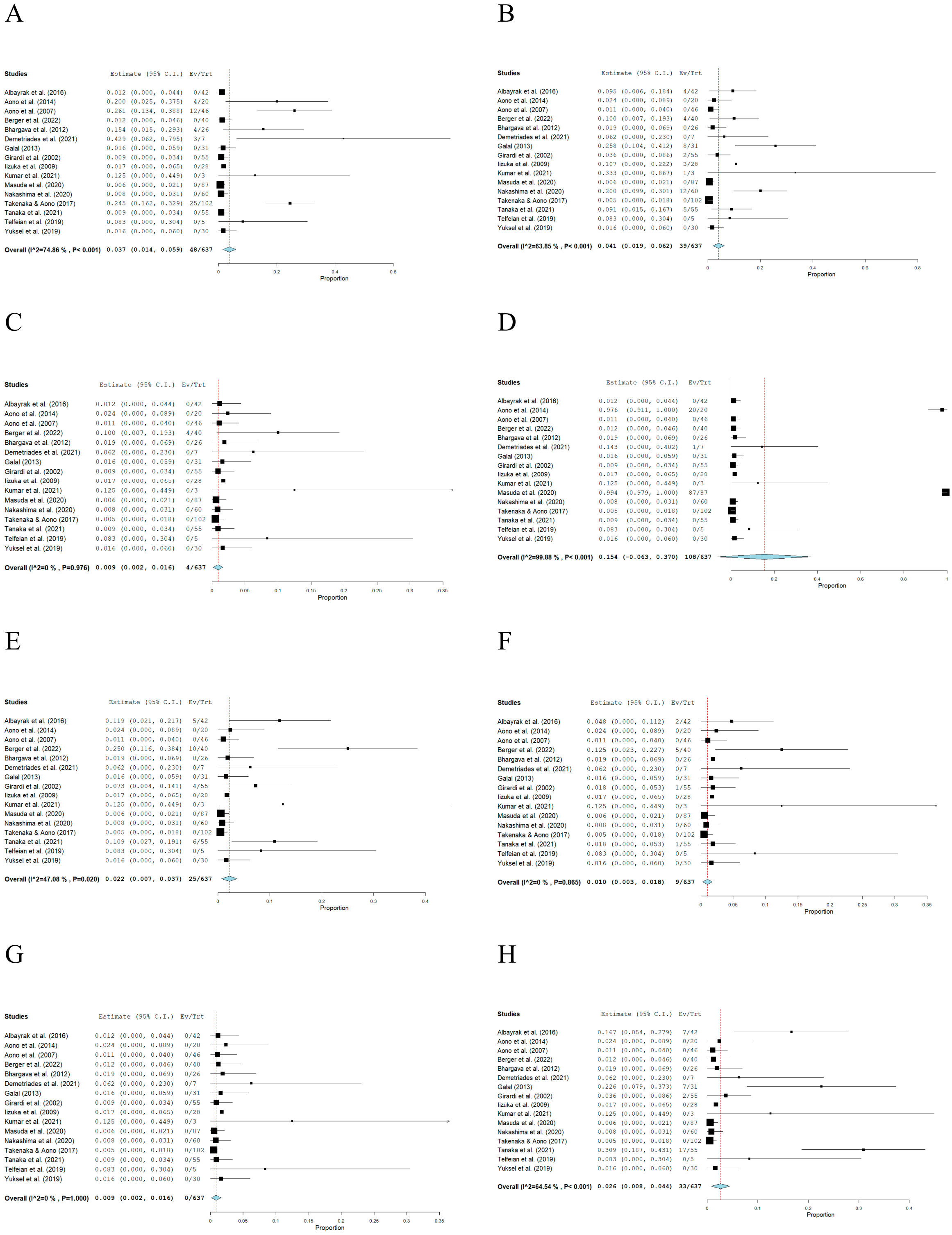
A
Studies Estimate (95% C.I.) Ev/Trt
Albayrak et al. (2016) 0.012 (0.000, 0.044) 0/42
Aono et al. (2014) 0.200 (0.025, 0.375) 4/20
Aono et al. (2007) 0.261 (0.134, 0.388) 12/46
Berger et al. (2022) 0.012 (0.000, 0.046) 0/40
Bhargava et al. (2012) 0.154 (0.015, 0.293) 4/26
Demetriades et al. (2021) 0.429 (0.062, 0.795) 3/7
Galal (2013) 0.016 (0.000, 0.059) 0/31
Girardi et al. (2002) 0.009 (0.000, 0.034) 0/55
Iizuka et al. (2009) 0.017 (0.000, 0.065) 0/28
Kumar et al. (2021) 0.125 (0.000, 0.449) 0/3
Masuda et al. (2020) 0.006 (0.000, 0.021) 0/87
Nakashima et al. (2020) 0.008 (0.000, 0.031) 0/60
Takenaka & Aono (2017) 0.245 (0.162, 0.329) 25/102
Tanaka et al. (2021) 0.009 (0.000, 0.034) 0/55
Telfeian et al. (2019) 0.083 (0.000, 0.304) 0/5
Yuksel et al. (2019) 0.016 (0.000, 0.060) 0/30
Overall (I^2=74.86 % , P< 0.001) 0.037 (0.014, 0.059) 48/637
0 0.2 0.4 0.6
Proportion
B
Studies Estimate (95% C.I.) Ev/Trt
Albayrak et al. (2016) 0.095 (0.006, 0.184) 4/42
Aono et al. (2014) 0.024 (0.000, 0.089) 0/20
Aono et al. (2007) 0.011 (0.000, 0.040) 0/46
Berger et al. (2022) 0.100 (0.007, 0.193) 4/40
Bhargava et al. (2012) 0.019 (0.000, 0.069) 0/26
Demetriades et al. (2021) 0.062 (0.000, 0.230) 0/7
Galal (2013) 0.258 (0.104, 0.412) 8/31
Girardi et al. (2002) 0.036 (0.000, 0.086) 2/55
Iizuka et al. (2009) 0.107 (0.000, 0.222) 3/28
Kumar et al. (2021) 0.333 (0.000, 0.867) 1/3
Masuda et al. (2020) 0.006 (0.000, 0.021) 0/87
Nakashima et al. (2020) 0.200 (0.099, 0.301) 12/60
Takenaka & Aono (2017) 0.005 (0.000, 0.018) 0/102
Tanaka et al. (2021) 0.091 (0.015, 0.167) 5/55
Telfeian et al. (2019) 0.083 (0.000, 0.304) 0/5
Yuksel et al. (2019) 0.016 (0.000, 0.060) 0/30
Overall (I^2=63.85 % , P< 0.001) 0.041 (0.019, 0.062) 39/637
0 0.2 0.4 0.6 0.8
Proportion
C
Studies Estimate (95% C.I.) Ev/Trt
Albayrak et al. (2016) 0.012 (0.000, 0.044) 0/42
Aono et al. (2014) 0.024 (0.000, 0.089) 0/20
Aono et al. (2007) 0.011 (0.000, 0.040) 0/46
Berger et al. (2022) 0.100 (0.007, 0.193) 4/40
Bhargava et al. (2012) 0.019 (0.000, 0.069) 0/26
Demetriades et al. (2021) 0.062 (0.000, 0.230) 0/7
Galal (2013) 0.016 (0.000, 0.059) 0/31
Girardi et al. (2002) 0.009 (0.000, 0.034) 0/55
Iizuka et al. (2009) 0.017 (0.000, 0.065) 0/28
Kumar et al. (2021) 0.125 (0.000, 0.449) 0/3
Masuda et al. (2020) 0.006 (0.000, 0.021) 0/87
Nakashima et al. (2020) 0.008 (0.000, 0.031) 0/60
Takenaka & Aono (2017) 0.005 (0.000, 0.018) 0/102
Tanaka et al. (2021) 0.009 (0.000, 0.034) 0/55
Telfeian et al. (2019) 0.083 (0.000, 0.304) 0/5
Yuksel et al. (2019) 0.016 (0.000, 0.060) 0/30
Overall (I^2=0 % , P=0.976) 0.009 (0.002, 0.016) 4/637
0 0.05 0.1 0.15 0.2 0.25 0.3 0.35
Proportion
D
Studies Estimate (95% C.I.) Ev/Trt
Albayrak et al. (2016) 0.012 (0.000, 0.044) 0/42
Aono et al. (2014) 0.976 (0.911, 1.000) 20/20
Aono et al. (2007) 0.011 (0.000, 0.040) 0/46
Berger et al. (2022) 0.012 (0.000, 0.046) 0/40
Bhargava et al. (2012) 0.019 (0.000, 0.069) 0/26
Demetriades et al. (2021) 0.143 (0.000, 0.402) 1/7
Galal (2013) 0.016 (0.000, 0.059) 0/31
Girardi et al. (2002) 0.009 (0.000, 0.034) 0/55
Iizuka et al. (2009) 0.017 (0.000, 0.065) 0/28
Kumar et al. (2021) 0.125 (0.000, 0.449) 0/3
Masuda et al. (2020) 0.994 (0.979, 1.000) 87/87
Nakashima et al. (2020) 0.008 (0.000, 0.031) 0/60
Takenaka & Aono (2017) 0.005 (0.000, 0.018) 0/102
Tanaka et al. (2021) 0.009 (0.000, 0.034) 0/55
Telfeian et al. (2019) 0.083 (0.000, 0.304) 0/5
Yuksel et al. (2019) 0.016 (0.000, 0.060) 0/30
Overall (I^2=99.88 % , P< 0.001) 0.154 (-0.063, 0.370) 108/637
0 0.2 0.4 0.6 0.8 1
Proportion
E
Studies Estimate (95% C.I.) Ev/Trt
Albayrak et al. (2016) 0.119 (0.021, 0.217) 5/42
Aono et al. (2014) 0.024 (0.000, 0.089) 0/20
Aono et al. (2007) 0.011 (0.000, 0.040) 0/46
Berger et al. (2022) 0.250 (0.116, 0.384) 10/40
Bhargava et al. (2012) 0.019 (0.000, 0.069) 0/26
Demetriades et al. (2021) 0.062 (0.000, 0.230) 0/7
Galal (2013) 0.016 (0.000, 0.059) 0/31
Girardi et al. (2002) 0.073 (0.004, 0.141) 4/55
Iizuka et al. (2009) 0.017 (0.000, 0.065) 0/28
Kumar et al. (2021) 0.125 (0.000, 0.449) 0/3
Masuda et al. (2020) 0.006 (0.000, 0.021) 0/87
Nakashima et al. (2020) 0.008 (0.000, 0.031) 0/60
Takenaka & Aono (2017) 0.005 (0.000, 0.018) 0/102
Tanaka et al. (2021) 0.109 (0.027, 0.191) 6/55
Telfeian et al. (2019) 0.083 (0.000, 0.304) 0/5
Yuksel et al. (2019) 0.016 (0.000, 0.060) 0/30
Overall (I^2=47.08 % , P=0.020) 0.022 (0.007, 0.037) 25/637
0 0.1 0.2 0.3 0.4
Proportion
F
Studies Estimate (95% C.I.) Ev/Trt
Albayrak et al. (2016) 0.048 (0.000, 0.112) 2/42
Aono et al. (2014) 0.024 (0.000, 0.089) 0/20
Aono et al. (2007) 0.011 (0.000, 0.040) 0/46
Berger et al. (2022) 0.125 (0.023, 0.227) 5/40
Bhargava et al. (2012) 0.019 (0.000, 0.069) 0/26
Demetriades et al. (2021) 0.062 (0.000, 0.230) 0/7
Galal (2013) 0.016 (0.000, 0.059) 0/31
Girardi et al. (2002) 0.018 (0.000, 0.053) 1/55
Iizuka et al. (2009) 0.017 (0.000, 0.065) 0/28
Kumar et al. (2021) 0.125 (0.000, 0.449) 0/3
Masuda et al. (2020) 0.006 (0.000, 0.021) 0/87
Nakashima et al. (2020) 0.008 (0.000, 0.031) 0/60
Takenaka & Aono (2017) 0.005 (0.000, 0.018) 0/102
Tanaka et al. (2021) 0.018 (0.000, 0.053) 1/55
Telfeian et al. (2019) 0.083 (0.000, 0.304) 0/5
Yuksel et al. (2019) 0.016 (0.000, 0.060) 0/30
Overall (I^2=0 % , P=0.865) 0.010 (0.003, 0.018) 9/637
0 0.05 0.1 0.15 0.2 0.25 0.3 0.35
Proportion
G
Studies Estimate (95% C.I.) Ev/Trt
Albayrak et al. (2016) 0.012 (0.000, 0.044) 0/42
Aono et al. (2014) 0.024 (0.000, 0.089) 0/20
Aono et al. (2007) 0.011 (0.000, 0.040) 0/46
Berger et al. (2022) 0.012 (0.000, 0.046) 0/40
Bhargava et al. (2012) 0.019 (0.000, 0.069) 0/26
Demetriades et al. (2021) 0.062 (0.000, 0.230) 0/7
Galal (2013) 0.016 (0.000, 0.059) 0/31
Girardi et al. (2002) 0.009 (0.000, 0.034) 0/55
Iizuka et al. (2009) 0.017 (0.000, 0.065) 0/28
Kumar et al. (2021) 0.125 (0.000, 0.449) 0/3
Masuda et al. (2020) 0.006 (0.000, 0.021) 0/87
Nakashima et al. (2020) 0.008 (0.000, 0.031) 0/60
Takenaka & Aono (2017) 0.005 (0.000, 0.018) 0/102
Tanaka et al. (2021) 0.009 (0.000, 0.034) 0/55
Telfeian et al. (2019) 0.083 (0.000, 0.304) 0/5
Yuksel et al. (2019) 0.016 (0.000, 0.060) 0/30
Overall (I^2=0 % , P=1.000) 0.009 (0.002, 0.016) 0/637
0 0.05 0.1 0.15 0.2 0.25 0.3 0.35
Proportion
H
Studies Estimate (95% C.I.) Ev/Trt
Albayrak et al. (2016) 0.167 (0.054, 0.279) 7/42
Aono et al. (2014) 0.024 (0.000, 0.089) 0/20
Aono et al. (2007) 0.011 (0.000, 0.040) 0/46
Berger et al. (2022) 0.012 (0.000, 0.046) 0/40
Bhargava et al. (2012) 0.019 (0.000, 0.069) 0/26
Demetriades et al. (2021) 0.062 (0.000, 0.230) 0/7
Galal (2013) 0.226 (0.079, 0.373) 7/31
Girardi et al. (2002) 0.036 (0.000, 0.086) 2/55
Iizuka et al. (2009) 0.017 (0.000, 0.065) 0/28
Kumar et al. (2021) 0.125 (0.000, 0.449) 0/3
Masuda et al. (2020) 0.006 (0.000, 0.021) 0/87
Nakashima et al. (2020) 0.008 (0.000, 0.031) 0/60
Takenaka & Aono (2017) 0.005 (0.000, 0.018) 0/102
Tanaka et al. (2021) 0.309 (0.187, 0.431) 17/55
Telfeian et al. (2019) 0.083 (0.000, 0.304) 0/5
Yuksel et al. (2019) 0.016 (0.000, 0.060) 0/30
Overall (I^2=64.54 % , P< 0.001) 0.026 (0.008, 0.044) 33/637
0 0.1 0.2 0.3 0.4
Proportion

I

J

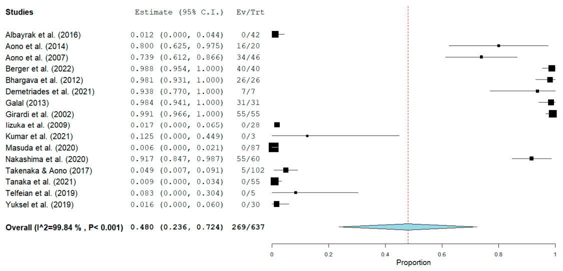

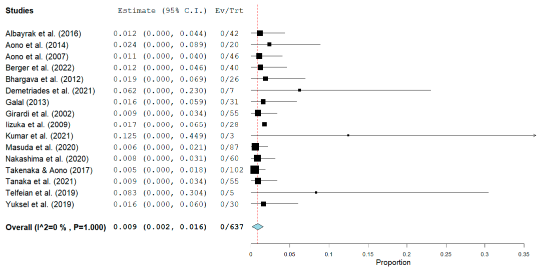

Supplementary Figure S7: Comorbidities and impairments. A) Cauda equina; B) Diabetes; C) Dyslipidaemia; D) Gluteus medius paralysis; E) Hypertension; F) Ischaemic heart disease; G) Osteoporosis; H) Other; I) Radiculopathy; J) Spinal trauma (2, 3, 8-10, 15, 19, 20, 22, 27-32).

A

B

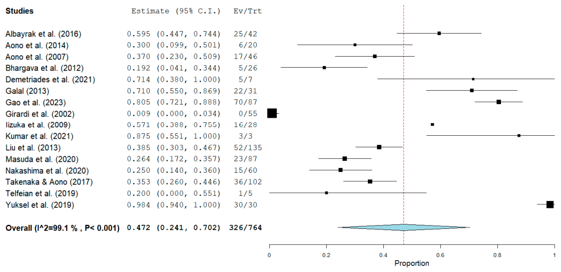

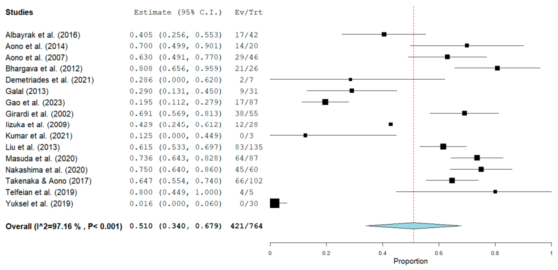

C

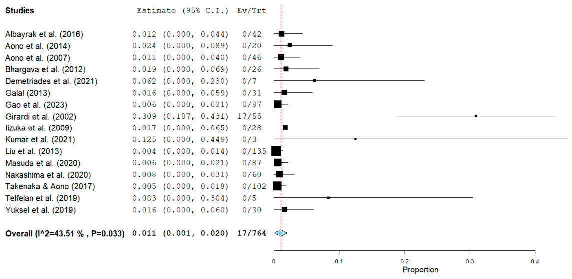

Supplementary Figure S8: Preoperative manual muscle test (MMT) scoring. A) Preoperative MMT score 0-1; B) Preoperative MMT score 2-3; C) Preoperative MMT score 4 (2, 4, 8, 10, 15-17, 19-22, 27, 29-32).

A

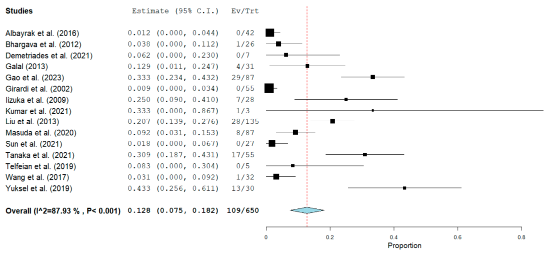

B

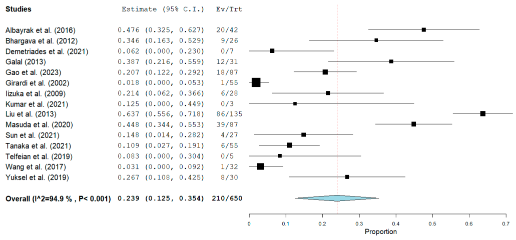

C

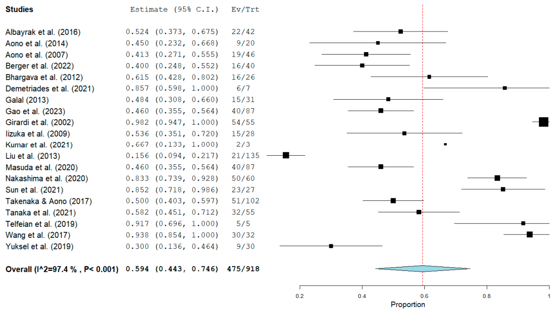

D

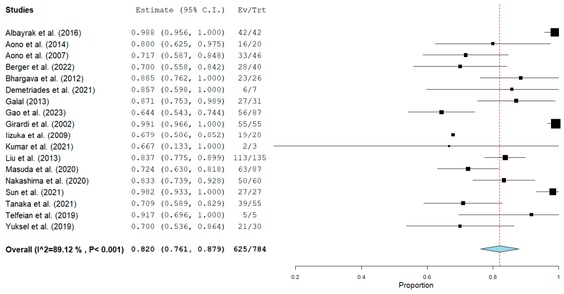

E

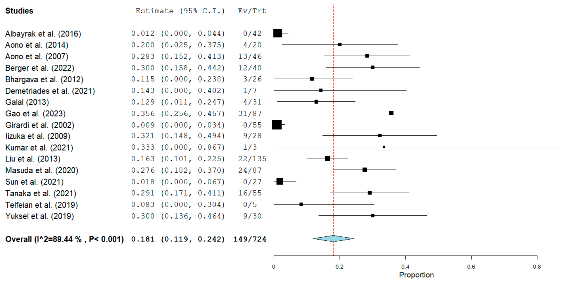

Supplementary Figure S9: Postoperative manual muscle test (MMT) scoring. A) Postoperative MMT score 0-1; B) Postoperative MMT score 2-3; C) Postoperative MMT score 4-5; D) Improvement MMT ≥ 1; E) No improvement (2-4, 8-10, 15-22, 27-32).

A

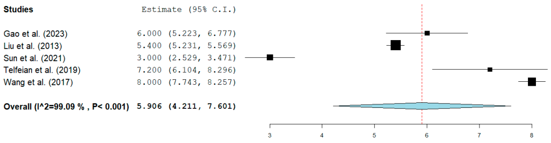

B

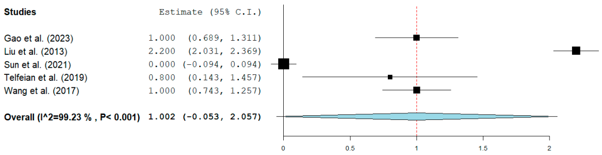

Supplementary Figure S10: Visual analogue scale (VAS). A) Mean preoperative VAS; B) Mean postoperative VAS (9, 16-19).

A

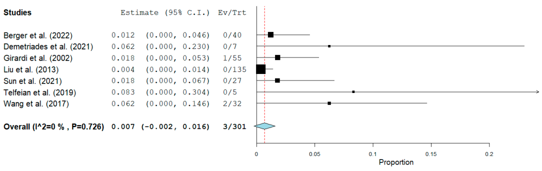

B

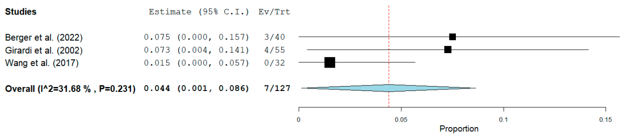

Supplementary Figure S11: Complications and reoperation. A) Overall complications; B) Reoperations (2, 9, 17-19, 21, 28).

A

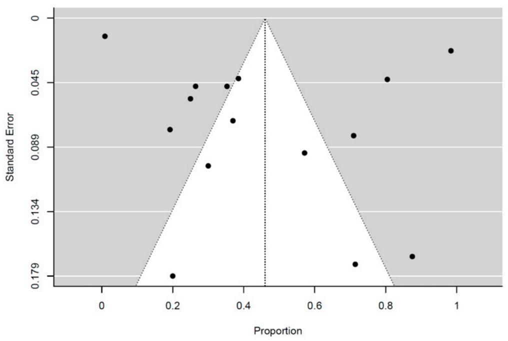

B

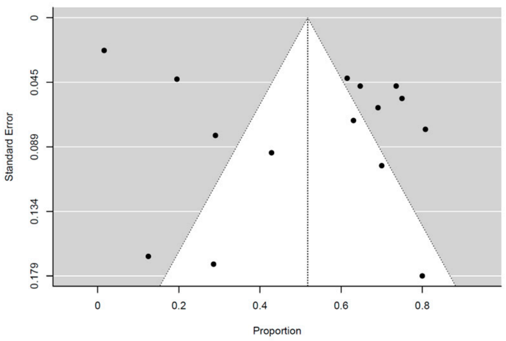

C

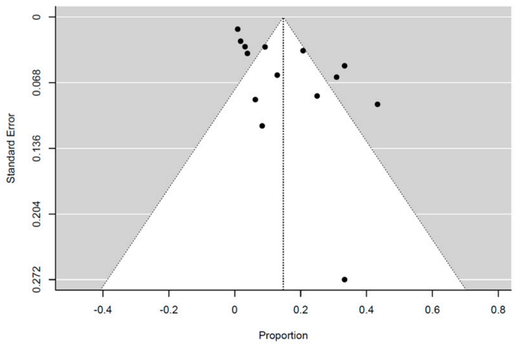

D

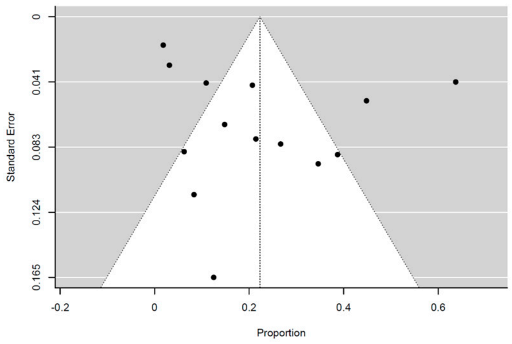

E

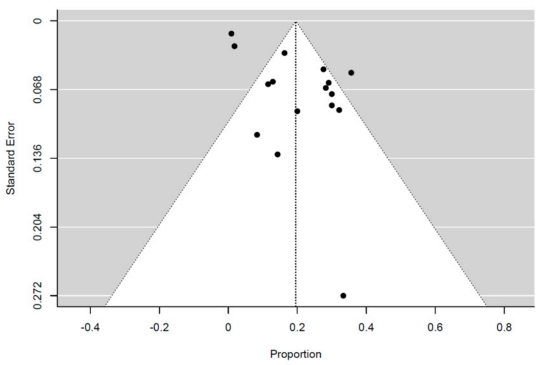

F

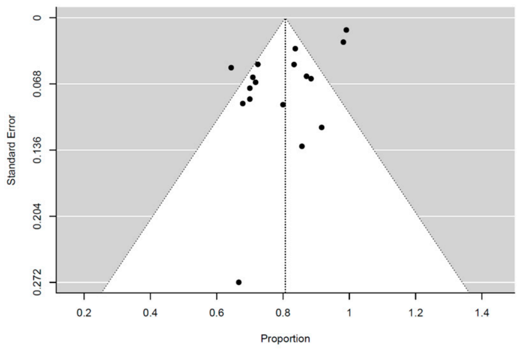

G

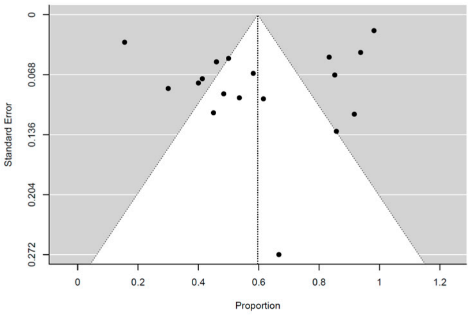

Supplementary Figure S12: Funnel plots. A) Pre-op 0-1; B) Pre-op 2-3; C) Post-op 0-1; D) Post-op 2-3; E) No improvement; F) Improvement; G) Post-op recovery 4-5 (2-4, 8-10, 15-22, 27-32)
